# Silencing of microRNA-146a alleviates the neural damage in temporal lobe epilepsy by down-regulating Notch-1

**DOI:** 10.1186/s13041-019-0523-7

**Published:** 2019-12-03

**Authors:** Hui Huang, Guiyun Cui, Hai Tang, Lingwen Kong, Xiaopeng Wang, Chenchen Cui, Qihua Xiao, Huiming Ji

**Affiliations:** 1 Department of Neurology, Huaibei People’s Hospital, No. 66, Huaihai West Road, Huaibei City, 235000 Anhui Province China; 2 Epilepsy Center, Affiliated Hospital, Xuzhou Medical University, No. 99, Huaihai West Road, Xuzhou City, 221002 Jiangsu Province China; 3 Medical Laboratory, Affiliated Hospital, Xuzhou Medical University, No. 99, Huaihai West Road, Xuzhou City, 221002 Jiangsu Province China

**Keywords:** Temporal lobe epilepsy, microRNA-146a, Neuronal damage, Notch-1, Caspase-9

## Abstract

This study aimed to evaluate the specific regulatory roles of microRNA-146a (miRNA-146a) in temporal lobe epilepsy (TLE) and explore the related regulatory mechanisms. A rat model of TLE was established by intraperitoneal injection of lithium chloride-pilocarpine. These model rats were injected intracerebroventricularly with an miRNA-146a inhibitor and Notch-1 siRNA. Then, neuronal damage and cell apoptosis in the cornu ammonis (CA) 1 and 3 regions of the hippocampus were assessed. SOD and MDA levels in the hippocampus were detected by chromatometry, and IL-1β, IL-6, and IL-18 levels were detected by ELISA. Then, we evaluated the expression levels of caspase-9, GFAP, Notch-1, and Hes-1 in the hippocampus. The interaction between Notch-1 and miRNA-146a was assessed by a dual luciferase reporter gene assay. A rat model of TLE was successfully established, which exhibited significantly increased miRNA-146a expression in the hippocampus. Silencing of miRNA-146a significantly alleviated the neuronal damage and cell apoptosis in the CA1 and CA3 regions of the hippocampus in TLE rats and decreased MDA, IL-1β, IL-6, and IL-18 levels and increased SOD levels in the hippocampus of TLE rats. In addition, silencing of miRNA-146a significantly decreased the expression levels of caspase-9, GFAP, Notch-1, and Hes-1 in the hippocampus of TLE rats. Notch-1 was identified as a target of miRNA-146a and silencing of Notch-1 aggravated the neuronal damage in the CA1 and CA3 regions. Silencing of miRNA-146a alleviated the neuronal damage in the hippocampus of TLE rats by down-regulating Notch-1.

## Background

Temporal lobe epilepsy (TLE) is a common, intractable form of epilepsy in adults that is characterized by recurrent, unprovoked focal seizures originating from the temporal lobe [1]. TLE mainly affects the medial temporal lobe and limbic network and involves the entorhinal cortex, amygdala, and hippocampus in the dentate gyrus and cornu ammonis (CA) regions [2]. Hippocampal sclerosis is frequently observed in TLE, along with neuron loss and gliosis [3]. Temporal lobe resection is the most effective therapeutic strategy for TLE, especially in drug-resistant cases [4]. Previous studies have shown that temporal lobe resection provides better seizure freedom and quality of life than prolonged drug therapy [5, 6]. Approximately 60–80% of patients who received an anterior temporal lobectomy achieved seizure freedom within 1–2 years, and approximately 50% of patients achieved durable seizure freedom within 10 years [7]. However, up to 40% of patients with refractory TLE continue to experience disabling postoperative seizures [5]. In addition, the outcomes of TLE patients who underwent surgery are limited by diverse postoperative neurological complications, such as visual field deficits, hemiparesis, dysphasia, cerebral ischemia, and cranial nerve paresis [8]. Therefore, novel, effective therapeutic strategies for TLE with fewer adverse effects are urgently needed.

MicroRNAs (miRNAs) are small noncoding RNAs that are involved in the post-transcriptional regulation of target mRNAs [9]. miRNAs play important regulatory roles in neuronal development and are involved in the occurrence and development of TLE [10]. Previous studies have shown that, in the hippocampus of TLE rats, miRNA-27a, − 54, − 210, − 345-3p, − 365-5p, − 423-3p, and − 455-3p are significantly up-regulated, and miRNA-33, −135b, − 138, − 221, − 222 and − 296-5p are significantly down-regulated [10–12]. These miRNAs have diverse regulatory roles in TLE. For example, up-regulation of miRNA-34a reduces neuronal apoptosis in the hippocampus of post-status epilepticus rats by inhibiting caspase-3 [10]. Down-regulation of miRNA-296-5p promotes neuronal apoptosis in the hippocampus of chronic TLE rats by activating caspase-3 [11]. It is worth noting that miRNA-146a, an miRNA that positively regulates inflammatory responses, is also up-regulated in TLE [13]. It has been reported that astroglial miRNA-146a expression is increased in regions of neuron loss and reactive gliosis in human TLE with hippocampal sclerosis [14]. miRNA-146a is up-regulated in both immature rats and children with mesial TLE, depending on the disease stage [15]. However, the specific roles of miRNA-146a in the pathological changes that occur in TLE are still not fully understood.

Notch signalling is a highly conserved cell signalling pathway that is involved in the regulation of diverse fundamental cellular processes, such as proliferation, stem cell maintenance, and differentiation [16]. Notch signalling not only maintains the self-renewal property and inhibits the neurogenesis of neural stem cells but also regulates subsequent lineage selection between the neuronal and glial cells in intermediate progenitors [17]. Notch signalling also plays a critical role in the development of TLE. It has been shown that Notch signalling is up-regulated in TLE mice and activation of Notch signalling further promotes the excitation of CA1 pyramidal neurons in acute seizures [18]. Notch-1 and hairy and enhancer of split 1 (Hes 1) are up-regulated in the temporal neocortex of patients with intractable TLE [19]. However, the regulatory mechanisms underlying the effects of miRNA-146a in Notch signalling remain unclear.

In this study, a rat model of TLE was established and injected with an siRNA targeting miRNA-146a siRNA to silence miRNA-146a expression. The effects of miRNA-146a down-regulation in TLE rats were evaluated in terms of neuronal damage, apoptosis, oxidative stress, and inflammatory responses. The regulatory mechanisms involving miRNA-146a related to caspase-9, glial fibrillary acidic protein (GFAP), and Notch-1 were also evaluated. Our findings revealed the inhibitory effects of miRNA-146a down-regulation on neural damage in TLE, and provided new insights into mechanisms that could be exploited in the treatment of TLE.

## Methods

### Establishment of a rat model of TLE

A total of 128 male Wistar rats (180–220 g, 6–8-week-old, SPF grade) were purchased from the Animal Experimental Center of the Hubei Center for Disease Control and Prevention (Wuhan, China). The rats were fed in a standalone environment at 22 °C and 50% relative humidity under a 12-h light/dark cycle, with free access to water and food. The rats were randomly divided into the control (*N* = 20) and model (*N* = 108) groups. TLE was induced in model group rats by an intraperitoneal injection of 3 mmol/kg lithium chloride (Shharvest, Shanghai, China), followed by 30 mg/kg pilocarpine (Shharvest) at 24 h post-lithium chloride treatment as previously described [20]. Then, 10 mg/kg pilocarpine was injected into the rats every 15 min (up to 5 times), until IV grade epilepsy was achieved as described by Racine [21]. At 1 h post-epilepsy induction, epilepsy was terminated by an intraperitoneal injection of 10 mg/kg diazepam (Shharvest). Twelve rats died due to uncontrolled epilepsy, and the remaining 96 TLE rats were considered to be successfully established and were used as the model group. Rats in the control group (*N* = 20) were injected with an equal amount of physiological saline. After the experiment, all surviving rats were sacrificed by decapitation. The ethics committee of Affiliated Hospital, Xuzhou Medical University approved the study, and all experiments performed were in accordance with the Guide for the Care and Use of Laboratory Animals published by the United States National Institutes of Health (Bethesda, MD, USA).

### Electroencephalogram (EEG) assay

Twenty-four hours post-epilepsy induction, all rats were anaesthetized with an intraperitoneal injection of 10% chloral hydrate and fixed on an operating floor. An electrode was connected to the scalp near the temporal lobe (0.65 cm in front of the porus acusticus internus, and 0.4 cm from the midline) as described previously [22]. A reference electrode was placed subcutaneously in the middle of the line between the outer canthus. The signal was band-pass filtered from 0.53 to 30 Hz, and input into a signal collection system. The number of spike-wave discharges (SWDs) during a 24-h period was recorded at 14 days after SE. EEG data were recorded, and epileptic seizures were regarded as high frequency (> 5 Hz) high amplitude (> 2× baseline) waves with polyspike discharges ≥5 s in duration.

### siRNA and miR-146a experiments

An siRNA targeting Notch-1 (siRNA Notch-1) and a negative control siRNA (siRNA NC) were synthesized by Shanghai GenePharma Company (Shanghai, China). After TLE was successfully induced, 10 μL of siRNA Notch-1 or siRNA NC mixed with 5 μL of Entranster™-in vivo (Engreen, Beijing, China) was injected intracerebroventricularly into rats in the siRNA Notch-1 and siRNA NC groups, respectively, (*N* = 16 in each group). For the miRNA-146a experiments, all rats were administered 0.8 μg of kainic acid by injection as previously described [23]. miRNA-146a expression in the rat hippocampus was antagonized or enhanced by injection of an miRNA-146a inhibitor (5′-AACCCAUGGAAUUCAGUUCUCA-3′) or miRNA-146a mimics (5′-UGAGAACUGAAUUCCAUGGGUU-3′), which were synthesized by Shanghai GenePharma Company. The miRNA-146a mimics, miRNA-146a inhibitor, miRNA-146a mimics negative control (miRNA-146a mimics NC, 5′-UUGUACUACACAAAAGUACUG-3′), and miRNA-146a inhibitor NC (5′-CAGUACUUUUGUGUAGUACAA-3′) were individually dissolved in normal saline at a concentration of 1 nmol/10 μl. Sixty-four rats were randomly divided into 4 groups (*N* = 16, each group) according to the molecules injected into the right lateral ventricle at AP = − 3.0 mm, L = − 2.2 mm, and V = − 2.8 mm from the Bregma. Each rat was injected with 1 nmol of miRNA-146a mimics, miRNA-146a inhibitor, or the corresponding NC, and 24 h later, the rats were subjected to kainic acid-induced status epilepticus. Three days after injection, the hippocampal tissues of six rats in each group were removed and analysed by haematoxylin-eosin (HE) staining, quantitative real-time PCR (qRT-PCR), immunohistochemistry (ICH), and western blotting. The 10 remaining rats in each group were sacrificed by decapitation, and the hippocampus tissues were collected and stored in liquid nitrogen.

### HE staining

Rats (*N* = 6, each group) were anaesthetized with an intraperitoneal injection of 10% chloral hydrate and fixed with 4% paraformaldehyde (Tianjin Institute of Chemical Reagents, Tianjin, China) through the auricula sinistra for 30 min. Then, the rats were sacrificed by decapitation, and the hippocampus tissues were removed and fixed in 4% paraformaldehyde for 3 h. After the tissues were paraffin-embedded, sliced (6 μm thick), and stained with HE, the pathological changes in the hippocampus (CA1 and CA3 regions) were observed under a microscope.

### qRT-PCR

Total RNA was extracted from the hippocampal tissues of rats (*N* = 5, each group) using TRIzol reagent (Thermo Fisher Scientific, Waltham, MA, USA) and reverse-transcribed using the FastQuant RT Kit (Tiangen, Beijing, China) according to the manufacturer’s instructions. qRT-PCR was performed with a Rotor-Gene 3000 (Corbett Research, Australia) using SuperReal PreMix Plus (SYBR Green; Tiangen). The following primers were used to detect miRNA-146a expression: miRNA-146a forward, 5′-TGAGAACTGAATTCCATGGGTT-3′ and miRNA-146a reverse, 5′-TGAGCTGAGAACTGAATTCCATG-3′. The PCR cycling program consisted of 95 °C for 3 min, followed by 40 cycles of 95 °C for 12 s and 62 °C for 40 s. U6 was amplified as an internal control with the following primers: U6 forward, 5′-CTCGCTTCGGCAGCACA-3′ and U6 reverse, 5′-AACGCTTCACGAATTTGCGT-3′. To detect Notch-1 expression, the following primers were used: Notch-1 forward, 5′-TCGCCGCAAGAGGCTTGAGATGCT-3′ and Notch-1 reverse, 5′-TCCGCTGCAGCACAGGCTTCA-3′. The PCR cycling program consisted of 95 °C for 10 min, followed by 40 cycles of 95 °C for 30 s and 60 °C for 60s. GAPDH was amplified as an internal control with the following primers: GAPDH forward, 5′-CCACCCATGGCAAATTCCATGGCA-3′ and GAPDH reverse, 5′-TCTAGACCGCAGGTCAGGTCCACC-3′. The relative expression levels of target genes were calculated using the 2^-ΔΔCt^ method [24].

### SOD, MDA, and cytokine assays

The hippocampal tissues of rats (*N* = 5, each group) were homogenized, and then centrifuged, and the supernatants were collected for analysis. SOD and MDA levels in the hippocampal tissues were detected by chromatometry, and cytokine levels (IL-1β, IL-6, and IL-18) were detected by enzyme linked immunosorbent assay (ELISA) using specific kits (Thermo Fisher Scientific) according to manufacturer’s instructions.

### Detection of apoptosis

The TUNEL assay was performed to detect apoptosis in the CA1 and CA3 regions of the hippocampus of TLE rats (*N* = 5, each group). Tissue sections of the CA1 and CA3 regions were stained with TUNEL (Roche, Basel, Switzerland) according to the manufacturer’s instructions. Three random regions of each section were observed under a microscope, and the number of positively stained cells (apoptotic cells) was counted.

### ICH

The expression levels of caspase-9 and GFAP in the hippocampal tissues of TLE rats (N = 5, in each group) were detected by ICH. Briefly, paraffin sections of hippocampal tissue were dewaxed and rehydrated with ethanol. Then, antigen retrieval was performed by incubation in 3% hydrogen peroxide for 10 min. Next, the sections were blocked with 5% bovine serum albumin (BSA) for 20 min, and incubated with primary antibodies (anti-caspase-9, 1:100, Epitomics, Burlingame, CA, USA; anti-GFAP, 1:100, Proteintech Group, Rosemont, IL, USA) overnight at 4 °C. After washing with PBS thrice, the sections were incubated with the secondary antibody (Wuhan Google, Wuhan, China) at 4 °C for 50 min. After staining with diaminobenzidine and haematoxylin, the tissue sections were observed under a microscope. The relative expression levels of caspase-9 and GFAP were quantitatively analysed with Image-Pro Plus 6.0 software.

### Western blotting

The hippocampal tissues of rats (*N* = 5, each group) were lysed in RIPA buffer containing 1 mM PMSF. Total proteins were separated by sodium dodecyl sulphate-polyacrylamide gel electrophoresis on 10% polyacrylamide gels and transferred to polyvinylidene fluoride membranes. After blocking with 5% BSA for 1 h, the membrane was incubated with primary antibodies (anti-Notch-1 and anti-Hes-1, 1:1000; Abcam, Cambridge, England) overnight at 4 °C. Then, the membrane was washed with TBST thrice and incubated with horseradish peroxidase-conjugated secondary antibody (1:1000; Cwbiotech, Beijing, China) for 2 h at 25 °C. Protein bands were visualized using a Gel imaging system (Thermo Fisher Scientific).

### Dual luciferase reporter (DLR) assay

The miRNA-target interaction between miRNA-146a and Notch-1 was assessed by a DLR assay. Briefly, 293 T cells (GeneChem, Shanghai, China) were seeded in 24-well plates and co-transfected with luciferase plasmids carrying wildtype Notch-1 (Notch-1-WT) or mutant Notch-1 (Notch-1-MT) (Ribobio, Guangzhou, China) and either miRNA-146a or a miRNA-146a inhibitor. After 48 h of incubation, the transfected cells were lysed in Passive Lysis Buffer, and then Luciferase Assay Regent and Stop & G10 reagents were sequentially added according to the manufacturer’s instructions (Thermo Fisher Scientific). The fluorescence intensity was detected with a Microplate Reader (Thermo Fisher Scientific).

### Statistical analyses

All experiments were performed in triplicate, and all data are expressed as the mean ± standard deviation (SD). Statistical analyses were performed using SPSS version 17.0 (SPSS Inc., Chicago, IL, USA). Comparisons between groups were performed using the *t* test (two groups) or one-way ANOVA followed by Fisher’s LSD post-hoc test (more than two groups). Spearman’s rank correlation analysis was performed to analyse the correlation between miRNA-146a and Notch-1. A *P*-value less than 0.05 was considered to be significant.

## Results

### TLE was successfully induced in rats

**Fig. 1:**
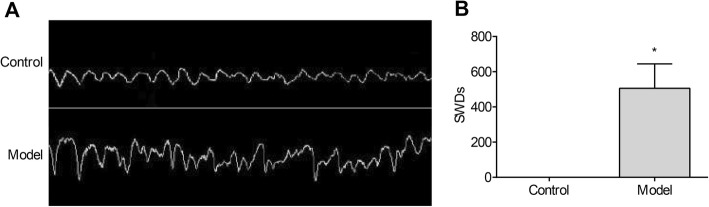
The electroencephalograms (EEGs) of rats were measured after 24 h post-epilepsy occurrence. Model, temporal lobe epilepsy (TLE) rats; Control, normal rats. **a** EEGs; **b**) the number of spike-and-wave discharges (SWDs). *, *P* < 0.05 vs. Control group

A rat model of TLE was established by intraperitoneal injection of lithium chloride-pilocarpine. Rats in the model group displayed obvious symptoms of TLE, including salivation, conjunctival congestion, nodding, blinking, chewing, and wet dog shakes, as well as falling and spinning at the later stage. In addition, EEG showed that the TLE model rats exhibited obvious epileptiform discharges, including high amplitude spikes and sharp waves, and spiked slow complex waves (Fig. 1a). In contrast, no epileptiform discharges were observed control group rats. The number of SWDs was significantly higher in the model group than in the control group (*P* < 0.05; Fig. 1b).

### miRNA-146a was up-regulated in the hippocampus of TLE rats

**Fig. 2:**
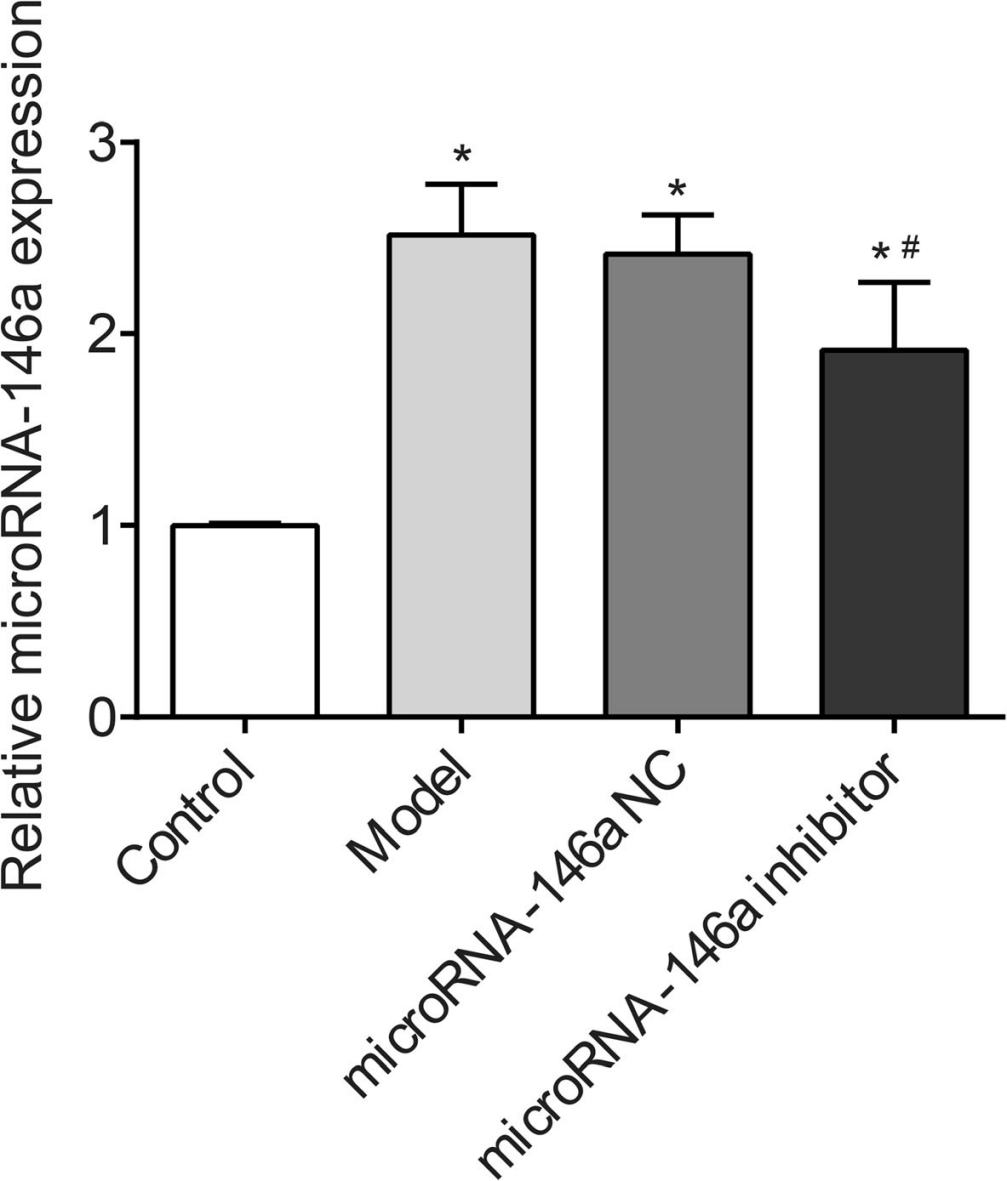
The expression of microRNA-146a (miRNA-146a) in hippocampus of rats detected by quantitative real-time PCR (qRT-PCR) (*N* = 8) at 3 days after the injection. Model, temporal lobe epilepsy (TLE) rats; Control, normal rats. *, P < 0.05 vs. Control group; #, P < 0.05 vs. Model group

miRNA-146a expression was detected in the hippocampus of rats by qRT-RCR, which showed that miRNA-146a expression was significantly higher in the model group than in the control group (*P* < 0.05). Injection of the miRNA-146a inhibitor significantly decreased the expression of miRNA-146a in the model group (*P* < 0.05). No significant difference in miRNA-146a expression was observed between the model and miRNA-146a NC groups (Fig. 2).

### Silencing of miRNA-146a alleviated the neuronal damage in the CA1 and CA3 regions of the hippocampus in TLE rats

**Fig. 3:**
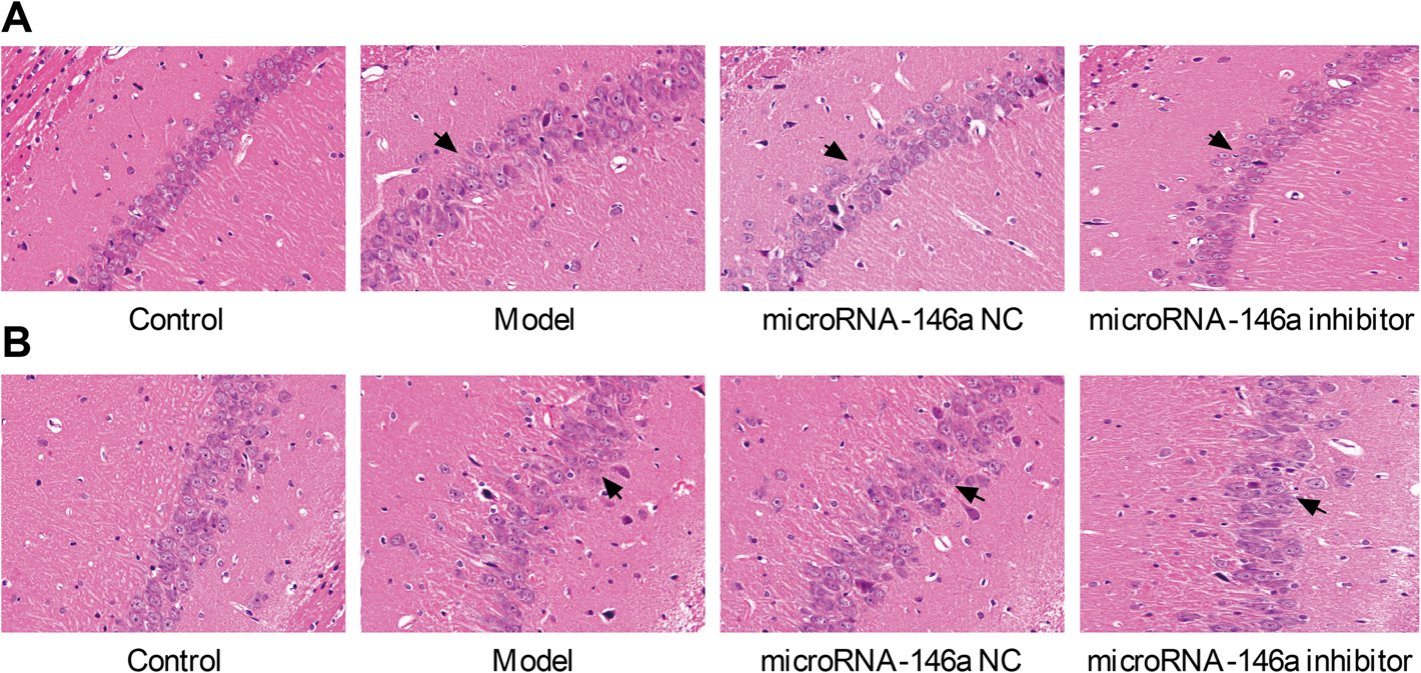
Pathological morphology of cornu ammonis (CA)1 and CA3 regions in hippocampus of rats were detected by hematoxylin-eosin (HE) staining (× 400) (N = 8) at 3 days after the injection. Model, temporal lobe epilepsy (TLE) rats; Control, normal rats; microRNA-146a (miRNA-146a) siRNA, TLE rats transfected with miRNA-146a siRNA; miRNA-146a negative control (miRNA-146a NC), TLE rats transfected with miRNA-146a NC. **a** CA1 region; **b**) CA3 region. Arrows represented the accumulation area of neurons

The pathological changes in the hippocampus of TLE rats were evaluated by HE staining. In the control group, neurons with normal morphology were arranged in well-defined layers in the CA1 and CA3 regions of the hippocampus, and no neuronal damage was observed (Fig. 3). In the model group, obvious neuron loss was observed in the CA1 and CA3 regions of the hippocampus. The neurons of rats in the model group were disordered, with abnormal structures and unclear boundaries. When compared to the model group, rats injected with the miRNA-146a inhibitor showed significantly reduced neuronal damage in the CA1 and CA3 regions of the hippocampus. The neuronal damage in the CA1 and CA3 regions of the hippocampus was not significantly affected by miRNA-146a NC injection (Fig. 3).

### Silencing of miRNA-146a reduced MDA levels and increased SOD levels in the hippocampus of TLE rats

**Fig. 4:**
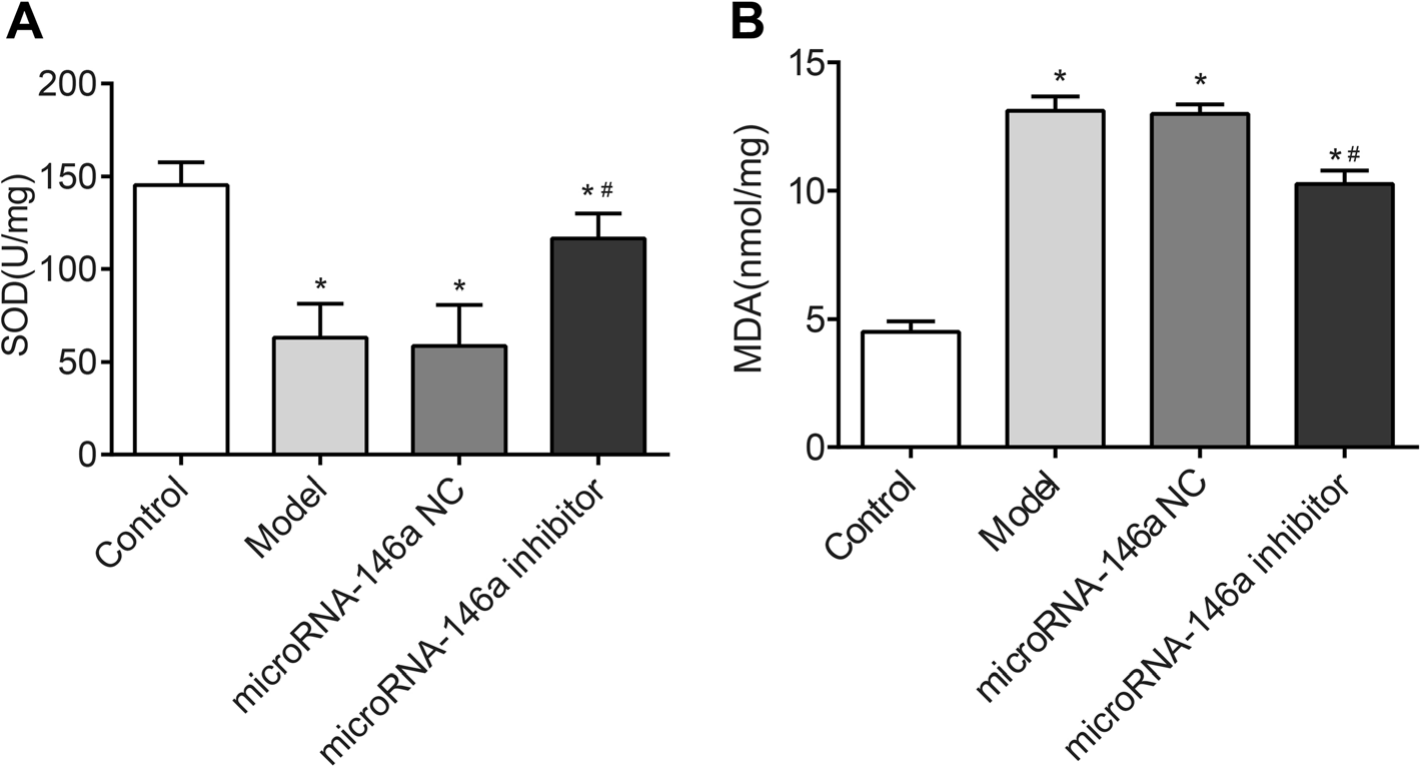
The levels of SOD and MDA in hippocampus of rats were detected by chromatometry (*N* = 8) at 3 days after the injection. Model, temporal lobe epilepsy (TLE) rats; Control, normal rats; microRNA-146a (miRNA-146a) siRNA, TLE rats transfected with miRNA-146a siRNA; miRNA-146a negative control (miRNA-146a NC), TLE rats transfected with miRNA-146a NC. **a** SOD level; **b**) MDA level. *, *P* < 0.05 vs. Control group; #, *P* < 0.05 vs. Model group

To explore the oxidative stress response of TLE rats, the levels of SOD and MDA in the hippocampus were detected by chromatometry. As shown in Fig. 4, significantly higher MDA levels and lower SOD levels in the hippocampus were detected in the model group than in the control group (*P* < 0.05). Injection of miRNA-146a siRNA significantly decreased MDA and increased SOD in the hippocampus of the model group (*P* < 0.05). No significant differences in SOD and MDA levels were detected between the model and miRNA-146a NC groups (Fig. 4).

### Silencing of miRNA-146a reduced IL-1β, IL-6, and IL-18 levels in the hippocampus of TLE rats

**Fig. 5:**
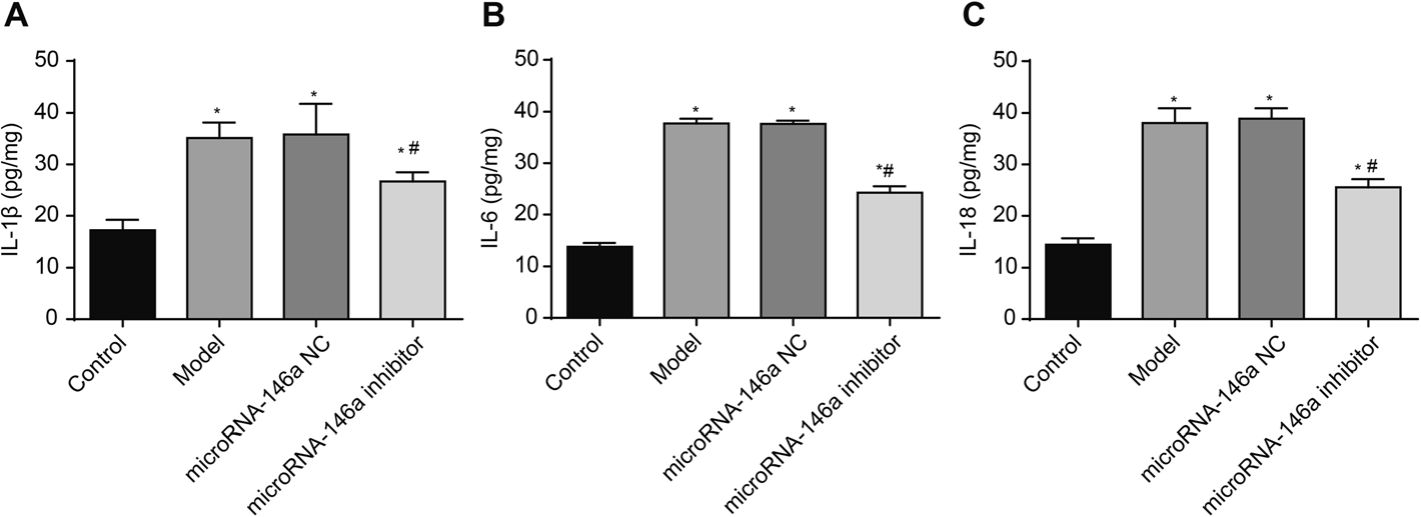
The levels of IL-1β, IL-6, and IL-18 in hippocampus of rats were detected by enzyme linked immunosorbent assay (ELISA) (*N* = 8) at 3 days after the injection. Model, temporal lobe epilepsy (TLE) rats; Control, normal rats; microRNA-146a (miRNA-146a) siRNA, TLE rats transfected with miRNA-146a siRNA; miRNA-146a negative control (miRNA-146a NC), TLE rats transfected with miRNA-146a NC. **a** IL-1β; **b**) IL-6; **c**) IL-18. *, *P* < 0.05 vs. Control group; #, *P* < 0.05 vs. Model group

To explore the inflammatory responses in TLE rats, the levels of IL-1β, IL-6, and IL-18 in the hippocampus were detected by ELISA. As shown in Fig. 5, the levels of IL-1β, IL-6, and IL-18 in the hippocampus were significantly higher in the model group than in the control group (*P* < 0.05). Injection of a miRNA-146a siRNA significantly decreased the levels of IL-1β, IL-6, and IL-18 in the hippocampus of the model group (*P* < 0.05). No significant differences *in* the levels of IL-1β, IL-6, and IL-18 were detected between the model and miRNA-146a NC groups (Fig. 5).

### Silencing of miRNA-146a inhibited cell apoptosis in the CA1 and CA3 regions of the hippocampus in TLE rats

**Fig. 6:**
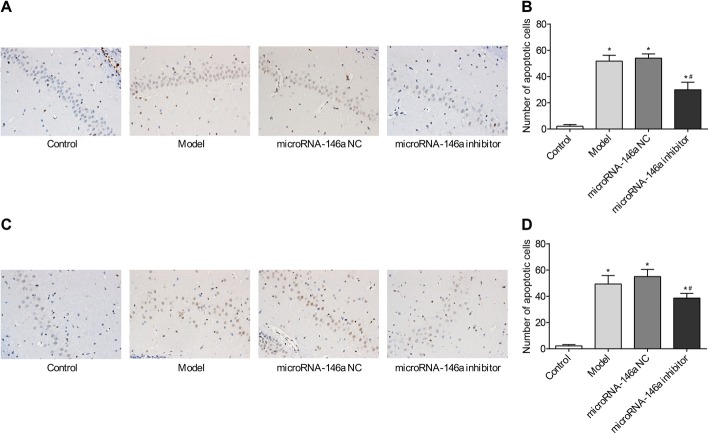
Cell apoptosis in CA1 and CA3 regions of hippocampus in rats detected by TUNEL (*N* = 5). Model, temporal lobe epilepsy (TLE) rats; Control, normal rats; microRNA-146a (miRNA-146a), TLE rats transfected with miRNA-146a siRNA; miRNA-146a negative control (miRNA-146a NC), TLE rats transfected with miRNA-146a NC. **a** microscopic observation of apoptotic cells in CA1 region; **b**) the number of apoptotic cells in stratum pyramidale of CA1 region; **c**) microscopic observation of apoptotic cells in CA3 region; **d**) the number of apoptotic cells in stratum pyramidale of CA3 region. *, *P* < 0.05 vs. Control group; #, *P* < 0.05 vs. Model group

Apoptosis of cells in the CA1 and CA3 regions of the hippocampus was detected by TUNEL assay. As shown in Fig. 6, the number of apoptotic cells in the CA1 and CA3 regions was significantly higher in the model group than in the control group (*P* < 0.05). Injection of miRNA-146a siRNA significantly decreased the number of apoptotic cells in the CA1 and CA3 regions of the model group (*P* < 0.05). No significant differences in apoptosis in the CA1 and CA3 regions were detected between the model and miRNA-146a NC groups (Fig. 6).

### Silencing of miRNA-146a decreased the expression of caspase-9 and GFAP in the hippocampus of TLE rats

**Fig. 7:**
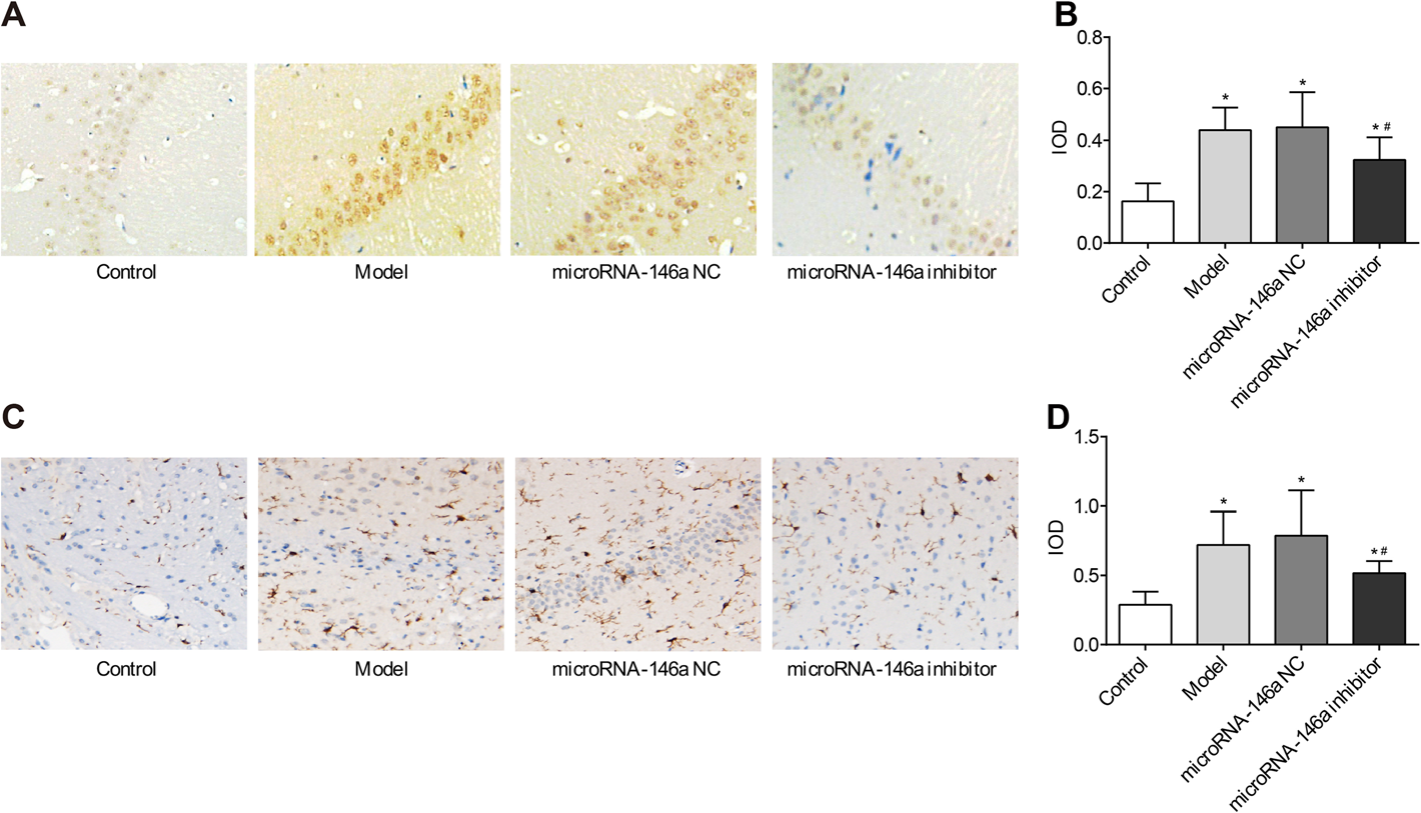
The expression of caspase-9 and GFAP in hippocampus was detected by immunohistochemistry (ICH) (× 400) (*N* = 8) at 3 days after the injection. Model, temporal lobe epilepsy (TLE) rats; Control, normal rats; microRNA-146a (miRNA-146a), TLE rats transfected with miRNA-146a siRNA; miRNA-146a negative control (miRNA-146a NC), TLE rats transfected with miRNA-146a NC. **a** microscopic observation of caspase-9-positive cells; **b**) the expression level of caspase-9; **c**) microscopic observation of GFAP-positive cells; **d**) the expression level of GFAP. *, *P* < 0.05 vs. Control group; #, *P* < 0.05 vs. Model group

Caspase-9 is an initiator of intrinsic apoptotic cell death [25], and GFAP expression is involved in regulating glial cell activation and apoptotic cell death [26]. To determine the role of miRNA-146a in TLE rats, the expression levels of caspase-9 and GFAP in model rats were assessed by ICH. As shown in Fig. 7, more cells in the hippocampus were positive for caspase-9 and GFAP in the model group than in the control group (*P* < 0.05). Injection of miRNA-146a siRNA significantly decreased the expression levels of caspase-9 and GFAP in the hippocampus of the model group (*P* < 0.05). No significant differences in the expression levels of caspase-9 and GFAP were observed between the model and miRNA-146a NC groups (Fig. 7).

### A miRNA-146a inhibitor increased Notch-1 and Hes-1 expression and decreased caspase-9 expression

**Fig. 8:**
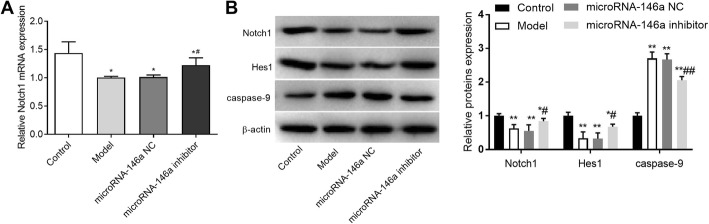
The expression of Notch-1, Hes-1 and caspase-9 in hippocampus detected by quantitative real-time PCR (qRT-PCR) and/or Western blot (*N* = 8) was performed at 3 days after the injection. Model, temporal lobe epilepsy (TLE) rats; Control, normal rats; microRNA-146a (miRNA-146a), TLE rats transfected with miRNA-146a siRNA; miRNA-146a negative control (miRNA-146a NC), TLE rats transfected with miRNA-146a NC. **a** the expression of Notch-1 detected by qRT-PCR (mRNA level); **b**) the expression of Notch-1, Hes-1 and caspase-9 detected by Western blot (protein level). *, *P* < 0.05 vs. Control group; **, *P* < 0.01 vs. Control group; #, P < 0.05 vs. Model group

The Notch signalling pathway has been implicated in the regulation of microglial activation and inflammatory-related neuronal injury in TLE model rats [27]. Hes-1 is the downstream effector of Notch. To determine the influence of miRNA-146a on Notch-1, Hes-1, and caspase-9 expression, qRT-PCR and western blotting were performed. As shown in Fig. 8a, b, Notch-1 mRNA and protein expression levels were significantly lower in the model group than in the control group (*P* < 0.01). Significantly lower Hes-1 protein expression was also observed in the model group when compared to that in the control group (*P* < 0.01). Conversely, caspase-9 was significantly higher in the model group than in the control group. In addition, a miRNA-146a inhibitor significantly increased Notch-1 and Hes-1 expression and decreased caspase-9 expression in the hippocampus of TLE rats (*P* < 0.05) (Fig. 8b). No significant differences in the expression levels of Notch-1, Hes-1, and caspase-9 were observed between the model and miRNA-146a NC groups (Fig. 8).

### Notch-1 is a target of miRNA-146a

**Fig. 9:**
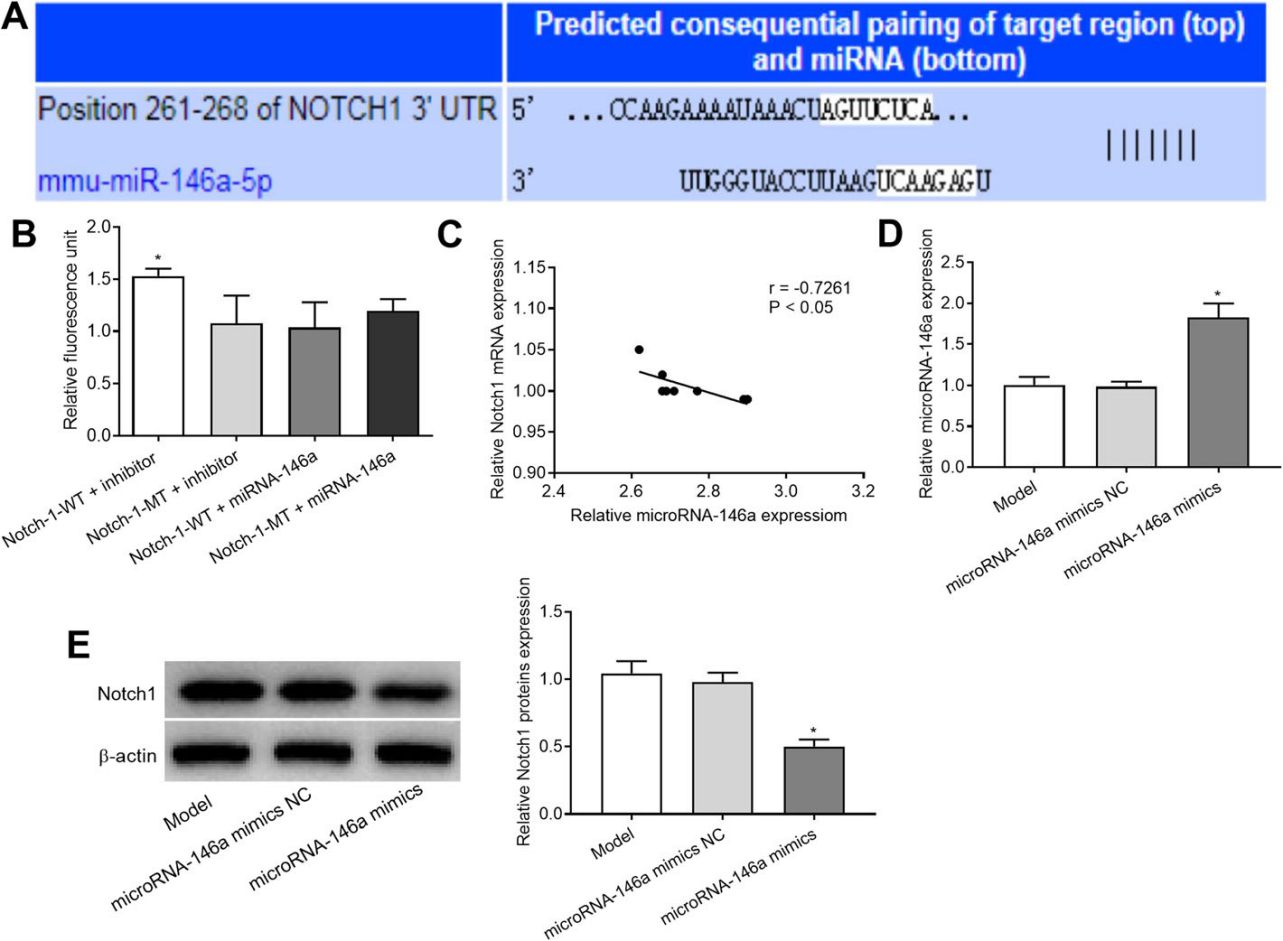
The interaction between Notch-1 and microRNA-146a (miRNA-146a). **a** a binding site of miRNA-146a at 3′-UTR of Notch-1 predicted by Target Scan; **b**) the relative fluorescence unit of co-transfected cells detected by dual luciferase reporter gene (DLR) assay. *, P < 0.05 vs. Notch-1-WT + miRNA-146a inhibitor. **c** Spearman’s rank correlation analysis was used to detect the correlation between Notch1 and miRNA-146a expression. **d** The expression of miRNA-146a transfected with miRNA-146a mimics or miRNA-146a mimics NC in the hippocampus of rats was detected by qRT-PCR. **e** Western blot analysis was used to detect the expression of Notch1 protein after transfected with miRNA-146a mimics or miRNA-146a mimics NC in the hippocampus of rats. *, *P* < 0.05 vs. miRNA-146a mimics NC

Online target gene prediction software (Target Scan) identified a binding site for miRNA-146a in the 3′-UTR of the Notch-1 gene (Fig. 9a). Then, the specific interaction between Notch-1 and miRNA-146a was evaluated by a DLR assay. As shown in Fig. 9b, cells co-transfected with Notch-1-WT + inhibitor exhibited significantly higher fluorescence intensity than cells co-transfected with Notch-1-WT + miRNA-146a, Notch-1-MT + miRNA-146a, and Notch-1-MT + inhibitor (*P* < 0.05; Fig. 9b). In addition, miRNA-146a expression was negatively associated with Notch-1 expression (r = − 0.7261, *P* < 0.05, Fig. 9c). To further confirm the effect of miRNA-146a on Notch-1 expression, we detected Notch1 protein expression in the hippocampus of rats transfected with miRNA-146a mimics or NC mimics. The transfection efficiency of the miRNA-146a mimics was verified when we found that the miRNA-146a mimics significantly increased the expression levels of miRNA-146a (Fig. 9d). Western blotting revealed that Notch-1 protein expression was lower in the hippocampus of rats injected with the miRNA-146a mimic than in rats injected with the NC mimic (*P* < 0.05, Fig. 9e).

### Silencing of Notch-1 aggravated the neuronal damage in the CA1 and CA3 regions of the hippocampus in TLE rats

**Fig. 10:**
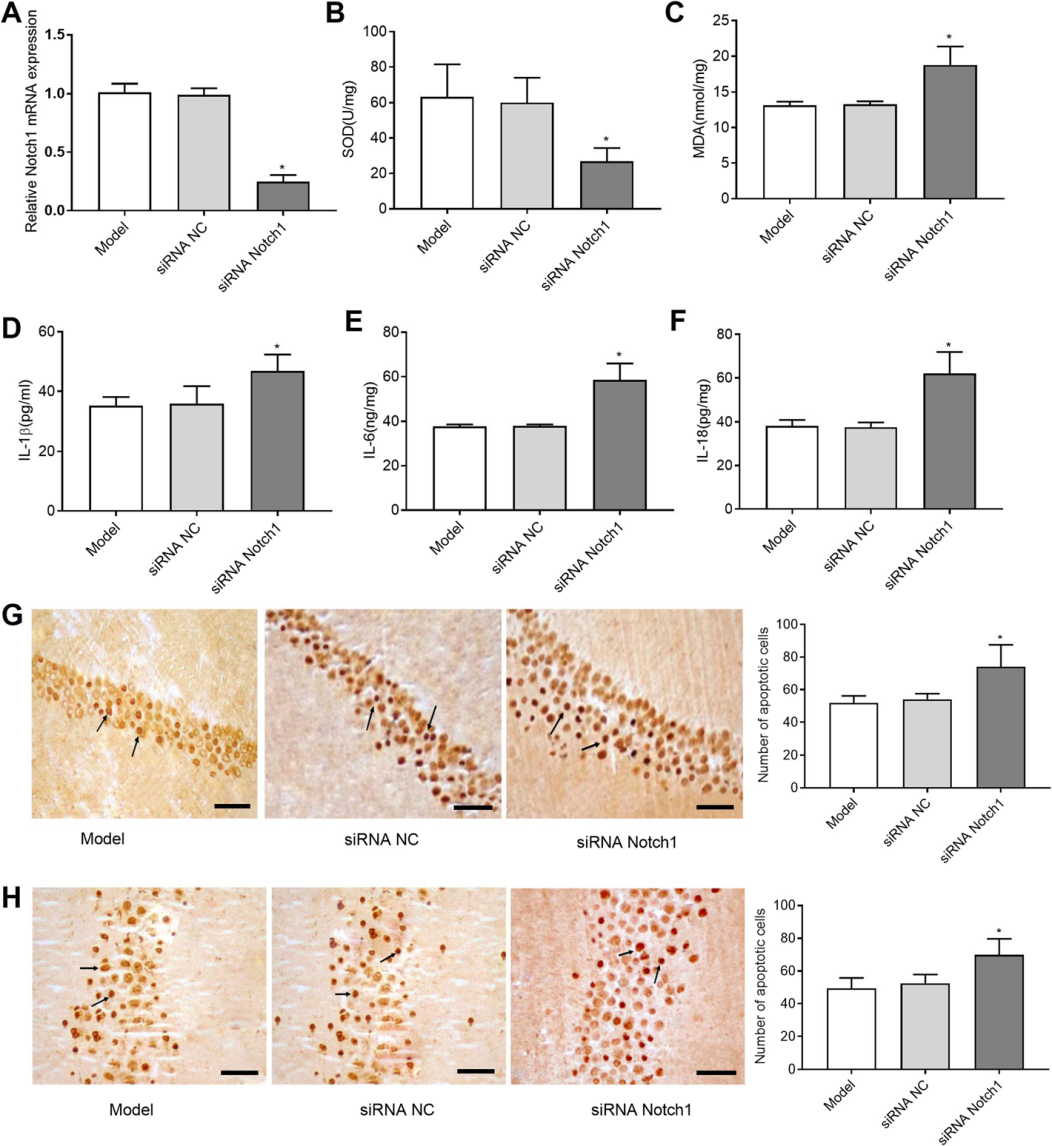
Silence Notch1 aggravated the severity of epileptic brain damage. **a** Rats (*N* = 8 in each group) were injected intracerebroventricularly with siRNA negative control (siRNA NC) or siRNA targeting Notch1 (siRNA Notch1) for three days and relative Notch1 expression was assessed by qRT-PCR to detect the siRNA efficiency against Notch1. After transfection of 3 days, the levels of SOD (**b**), MDA (**c**), IL-1β (**d**), IL-6 (**e**) and IL-18 (**f**) were detected. *P < 0.05 vs. siNC group. Photomicrographs of apoptotic cells in CA1 region (**g**) and (**h**) CA3 region (arrows indicate the positive cells, bar = 100 μm, magnification × 400)

To investigate the role of Notch-1 in the neuronal damage in the CA1 and CA3 regions of the hippocampus in TLE rats, the silencing efficiency of an siRNA directed against Notch1 or NC siRNA was assessed 3 days after intracerebroventricular injection. As shown in Fig. 10a, injection of siRNA Notch-1 significantly decreased the mRNA expression of Notch1 (*P* < 0.05). We then assessed the role of Notch-1 silencing on the neuronal damage in TLE rats and found that the levels of MDA, IL-1β, IL-6, and IL-18 were increased and the levels of SOD were decreased in rats in the siRNA Notch-1 group compared with the levels in rats of the siRNA NC group (P < 0.05, Fig. 10b-f). Additionally, silencing of Notch-1 increased the number of apoptotic cells in the CA1 and CA3 regions (P < 0.05, Fig. 10g, h). Taken together, these results suggested that silencing of Notch-1 aggravated the neuronal damage in the CA1 and CA3 regions of the hippocampus in TLE rats.

## Discussion

Epileptic animal models are often used to study the pathological changes in TLE as well as the therapeutic effects of anti-epileptic drugs [28]. In this study, a rat model of TLE was established by intraperitoneal injection of lithium chloride-pilocarpine. Although 12 rats died due to uncontrolled epilepsy, TLE was successfully induced in the remaining 48 TLE rats. Rats in the model group exhibited obvious TLE symptoms of at least grade IV as described by Racine. In addition, a significantly greater number of SWDs was observed in the model group than in the control group. These observations are consistent with the clinical characteristics of TLE in humans [29]. Since the pathological state of TLE can be simulated in the rat model of TLE, the specific roles of miRNA-146a in TLE were analysed in this study.

miRNA-146a is a critical regulator of multiple biological processes, including innate immunity, inflammatory responses, viral infection, and cancer [30]. Previous studies have shown that miRNA-146a is significantly up-regulated in both rats and humans with TLE [14, 15]. Consistent with previous studies, we found that miRNA-146a expression in the hippocampus was significantly higher in the model group than in the control group. The neuronal damage in TLE is characterized by obvious neuron loss in the CA1 and CA3 regions and the hilus of the dentate gyrus in the hippocampus [31]. In this study, obvious neuron loss was observed in the CA1 and CA3 regions of the hippocampus in TLE model rats. Knockdown of miRNA-146a with a targeting siRNA significantly inhibited the neuron loss in the CA1 and CA3 regions of the hippocampus. This indicates that silencing of miRNA-146a alleviates the neuron loss in the hippocampus of TLE rats. The inhibitory effect of miRNA-146a down-regulation on neuron loss in TLE is consistent with that of miRNA-199a-5p. A previous study demonstrated that knockdown of miRNA-199a-5p alleviates seizure-like EEG waves and protects against neuron loss in rats with epilepsy by up-regulating SIRT1 and subsequent deacetylating p53 [32]. We suspect that miRNA-146a may have a similar function to that of miRNA-199a-5p in rats with epilepsy.

Mitochondrial oxidative stress is closely associated with the occurrence and progression of TLE. The prolonged seizures in TLE result in the oxidation of cellular macromolecules, thereby contributing to neuron loss [33]. In this study, we found that injection of miRNA-146a siRNA significantly reduced MDA levels and increased SOD levels in the hippocampus of TLE rats. Our findings are consistent with a previous study on vitamin E. It has been reported that vitamin E pre-treatment significantly decreased MDA levels and increased SOD levels in the hippocampus of TLE rats, and this reduction in hippocampal oxidative stress decreased the severity and incidence of seizures and improved retrieval and recall in passive avoidance [34]. We suspect that silencing of miRNA-146a may alleviate the neural damage in the hippocampus of TLE rats by promoting ROS scavenging. The inflammatory response also plays an important role in TLE. Inflammatory cytokines, such as IL-1β, IL-6, IL-10, are involved in the pathophysiology of TLE by altering neuronal death and astrocytic activation [35–37]. In this study, we found that injection of miRNA-146a siRNA significantly decreased the levels of IL-1β, IL-6, and IL-18 in the hippocampus of TLE rats. Our findings are consistent with previous studies showing that miRNA-146a is positively correlated with the severity of inflammatory response [38, 39]. Silencing of miRNA-146a may reduce the inflammatory response by decreasing the production of inflammatory cytokines, thereby alleviating neural damage in the hippocampus of TLE rats.

Cell apoptosis in the hippocampus is a major manifestation of the neural damage in TLE. In this study, we found that the numbers of apoptotic cells in the CA1 and CA3 regions of the hippocampus were significantly higher in the model group than in the control group. Notably, injection of miRNA-146a siRNA in TLE rats significantly decreased the numbers of apoptotic cells. The inhibitory role of miRNA-146a down-regulation on cell apoptosis may be attributed to the mitigation of oxidative stress and inflammation. To further explore the molecular mechanisms underlying the role of miRNA-146a in TLE, the expression levels of caspase-9 and GFAP were measured in the hippocampus of TLE rats. The results showed that injection of miRNA-146a siRNA significantly inhibited the expression of caspase-9 and GFAP in the hippocampus of TLE rats. Caspase-9 is an initiator caspase that is involved in apoptosis and cytokine signalling. A previous study showed that caspase-9 is up-regulated in patients with medial TLE, and that caspase-9-activated caspase-3 is positively associated with the frequency of epileptic seizures in medial TLE [40]. Therefore, down-regulation of caspase-9 may contribute to the inhibition of cell apoptosis in the hippocampus of TLE rats. GFAP is a protoplasmic astrocyte marker that is up-regulated in patients with TLE [41]. GFAP levels reflect reactive gliosis, a prominent manifestation of TLE that involves structural and metabolic changes in astrocytes and microglia [42, 43]. Down-regulation of GFAP may also contribute to the inhibition of gliosis in the hippocampus of TLE rats. In summary, we suspect that silencing of miRNA-146a may inhibit apoptosis and gliosis in the hippocampus of TLE rats by down-regulating caspase-9 and GFAP.

Notch signalling plays an important role in the neuroinflammation and neuronal damage in TLE [27]. Numerous studies have demonstrated that Notch signalling is activated in both TLE model rats and humans with TLE [18, 19]. Consistent with previous studies, we found that the expression of Notch-1 and its downstream target gene Hes-1 were significantly higher in the model group than in the control group. In addition, administration of miRNA-146a siRNA significantly decreased the expression levels of Notch-1 and Hes-1 in the hippocampus of TLE rats. A previous study showed that Notch signalling is activated in response to seizure activity and contributes to the promotion of neuronal excitation in TLE [18]. We suspect that silencing of miRNA-146a may inhibit neuronal excitation in TLE rats by inhibiting Notch signalling. Additionally, we identified Notch-1 as a target of miRNA-146a. This finding further illustrates that the inhibitory role of miRNA-146a down-regulation in the neural damage in TLE is related to the inhibition of Notch signalling.

## Conclusions

In conclusion, silencing of miRNA-146a alleviated oxidative stress and the inflammatory response in TLE, thereby protecting against neuronal damage. The inhibitory effects of miRNA-146a on neuronal damage in TLE are associated with the inhibition of caspase-9, GFAP, and Notch-1. Therefore, down-regulation of miRNA-146a may be a useful therapeutic target in TLE. However, this study is still limited by the use of a rat model. Thus, the efficacy and safety of miRNA-146a down-regulation for the treatment of TLE is still unclear. Further research to identify novel drugs that specifically inhibit miRNA-146a is also needed.

## Data Availability

All data generated or analyzed during this study are included in this published article [and its supplementary information files].

## Abbreviations

DLR: Dual luciferase reporter gene
miRNA-146a: microRNA-146a
TLE: Temporal lobe epilepsy
